# Health Tract, No. 141

**Published:** 1865

**Authors:** 


HEALTH TRACT, No. 141.

### Changing Clothing.

It has come within the observation of many a reader that serious and severe illness has been induced, and even fatal sickness caused, by a change of clothing. Injury never comes, perhaps, by putting on more or warmer clothing, but by diminishing the amount inconsiderately. The first great general rule, and always the safest, is to make the change when you first dress in the morning; if you wait until you are uncomfortably warm during the day, it is most likely to be in the early part of the afternoon; in making the change then there are two or three causes of disease in operation; the fact of undressing endangers a check of perspiration; the garments about to be put on may not be perfectly dry, there may be no opportunity, even if they are dry, to warm them up to the heat of the body; and further, just about the time you have changed, the cool and damps of the afternoon and evening begin to come on, increasing until dark, while having been thrown off your guard by the warmth of the morning, you may not feel the necessity of a fire, and by tea-time you are surprised with a disagreeable chilliness running over you; then the cold has been taken, to settle in the eyes, causing weakness and watering; or in the head, giving a running at the nose, soiling a handkerchief in an hour; or in the throat, creating a raw or burning sensation at the little hollow at the bottom of the neck and top of the breast-bone; or on the covering of the lungs, to give the painful pleurisy; or in the lungs themselves, in the shape of a troublesome bronchitis, or a dangerous pneumonia; or in the bowels, causing weakening diarrhea; or on the covering of the bowels, inducing peritoneal inflammation, to end probably in death, in a few days.

It is very unsafe to lessen the amount of clothing sooner than the first of May, and then not in quality, but in less thickness of the same material; from yarn socks to worsted; from a thick, knitted flannel shirt to one of common woolen flannel; then, about the first of June, to a gauze flannel; if this is oppressive to some, then employ canton flannel. But it is certainly a great mistake for any body to wear any thing else next the skin, even in the hottest summer weather, than woolen flannel. Silk shirts next the skin can not be advocated on any tangible grounds; the moment a man begins to twaddle with you about "electrical influences," turn your heel upon him, and set him down as a presumptive and impudent ignoramus.

